# Using QuEChERS and HPLC Method to Monitor the Background Concentration of Polycyclic Aromatic Hydrocarbons in Commercial Black Tea Leaves and Infusions in Taiwan

**DOI:** 10.3390/toxics12020148

**Published:** 2024-02-14

**Authors:** Drewyan Minelly Harrison, Wei-Chung Chang, Hsin-Tang Lin

**Affiliations:** 1 International Master Program of Agriculture, National Chung Hsing University, Taichung 402-202, Taiwan; drewyanmharrison@gmail.com; 2 Graduate Institute of Food Safety, National Chung Hsing University, Taichung 402-202, Taiwan; weichung871227@gmail.com; 3 Department of Food Science and Biotechnology, National Chung Hsing University, Taichung 402-202, Taiwan; 4 Department of Law, National Chung Hsing University, Taichung 402-202, Taiwan

**Keywords:** *Camellia sinensis*, polycyclic aromatic hydrocarbons (PAHs), QuEChERS, high performance liquid chromatography (HPLC), limit of detection (LOD), limit of quantification (LOQ)

## Abstract

Tea is an integral part of Taiwanese culture and is a popular drink as it contains many beneficial compounds. However, during the processing of tea, polycyclic aromatic hydrocarbons (PAHs) may form. This study investigated the concentrations of PAH4 in different black tea leaves and tea infusions based on the origin of the tea. The samples were extracted using QuEChERS, while the content of PAH4 was analyzed by high performance liquid chromatography coupled to a fluorescence detector (HPLC-FLD). The content of PAH4 in the tea leaves ranged from 2.88 µg/kg to 218.2 µg/kg (dry weight), with the highest concentration being found in teas from Vietnam. The concentration of BaP ranged from ND to 47.92 µg/kg. The release of PAH4 from tea leaves to tea infusions was significantly low, with the highest transfer being 25.8%. In this study, all PAH4 compounds in commercial black tea leaves can be detected by QuEChERS extraction with a simple HPLC method.

## 1. Introduction

Polycyclic aromatic hydrocarbons (PAHs) are a large category of organic compounds containing two or more fused benzene rings [1]. These substances arise from the incomplete combustion of carbon-based materials [2]. PAHs are resistant to degradation, allowing them to persist in the environment for extended periods. Among the numerous PAHs, 16 have been identified by the European Food Safety Authority (EFSA) and are known as European Union (EU) priority PAHs [3]. The PAHs included in this list are benzo[a]pyrene (BaP), chrysene (CHR), benz[a]anthracene (BaA), benzo[b]fluoranthene (BbF), benzo[k]fluoranthene (BkF), benzo[ghi]perylene (BgP), dibenz[a,h]antracene (DhA), indeno[1,2,3-cd]pyrene (IcP), benzo[c]fluorene (BcL), benzo[j]fluoranthene (BjF), cyclopenta[c,d]pyrene (CPP), dibenzo[a,e]pyrene (DeP), dibenzo[a,h]pyrene (DhP), dibenzo[a,i]pyrene (DiP), dibenzo[a,l]pyrene (DlP), and and 5-methylchrysene (5-MC). In 2011, the EFSA identified the sum of BaP, BaA, BbF, and CHR (known as PAH4) as suitable indicators for the occurrence of PAHs in food.

Foods may become polluted with PAHs during the different stages of farming, as crops may take up PAHs present in soil, water, air or may become contaminated during the packaging process. Incomplete combustions could release PAHs and be spread in the air, water, sediments, soil and plants due to non-optimal temperature, oxygen content and high moisture [4,5]. High-temperature cooking processes such as grilling, smoking, frying, steaming, roasting and drying also play a role in the production of PAHs in food products [6]. So, as people consume these products, they may also consume PAHs. It is well known that smokers are exposed to large amounts of PAHs through cigarettes. For non-smokers, their exposure to PAHs mainly comes from their daily diet [5,7,8].

The analysis of toxic substance in food commodity is difficult due to the complexity of food matrices and trace concentrations of these compounds. QuEChERS (quick, easy, cheap, effective, rugged and safe) extraction has been developed and applied to extract PAHs from foodstuffs and commodities including meat [9,10,11,12], fish and seafood [9,13], milk [14], rice grain [15] and tea [16]. Gas chromatography–mass spectrometer (GC-MS) and HPLC-FLD are commonly used to analyze PAHs in foodstuffs. GC-MS was mostly used to analyze the U.S. Environmental Protection Agency (EPA) priority PAHs [11,17,18]. In recent years, HPLC-FLD was more commonly used to monitor the EU priority PAHs because of lower cost and easy operation [19,20].

Tea (*Camellia sinensis*) is a popular drink worldwide as it is known for its many health benefits. Tea contains many beneficial compounds such as phenolic acids, aromatic compounds, flavonoids, amino acids and alkaloids [21]. The main benefits of black teas are associated with polyphenols. Tea is an aromatic beverage which is prepared by brewing the processed leaves of the *Camellia sinensis* plant, which is a plant native to China, India and other east Asian countries. China and India are the top producers of tea in the world. Worldwide, the production of tea was estimated to be 6.5 million tons [22]. In 2021, the production of tea in Taiwan was only 11,900 tons [23]. As well producing its own tea, Taiwan also imports tea, with over 30,000 tons being imported yearly. Black tea accounts for more than 80% of all tea imports. The majority of black tea imports come from Vietnam and Sri Lanka. Vietnam is the largest exporter of tea to Taiwan, making up over a third of the imports. In 2021, Taiwan imported over 18,000 tons of tea from Vietnam [24]. Sri Lanka exports the second largest amount of tea to Taiwan, making up 15% of imports, followed by India, which makes up 10%. Taiwan also imports black tea from Indonesia, Kenya, Myanmar, and China, though in smaller amounts.

Black tea is a fully fermented tea and is one of the most popular types of tea around the globe. Along with most other teas, the final product depends on the manufacturing process. The manufacturing of black tea is a complex biological process, and the quality of the final product greatly depends on the composition of the fresh tea leaf, the extent to which the leaves are dehydrated, the extent to which they are broken down, and how much the leaves are fermented. Black tea production usually requires processes such as withering, rolling/cutting, and fermentation/oxidation to produce polyphenolic compounds and unique aroma and flavor. Then, drying is carried out to reduce the moisture content to less than 5% for packaging, transportation and storage [25]. Common drying methods include oven drying, sunlight drying, or drying on a temperature-controlled indoor floor. The typical drying temperature ranges from 80 to 90 °C by commercial dryers. The drying temperature of tea leaves could be adjusted to 90–120 °C or higher by charcoal, wood or other fuel firing to generate heat, especially in some tea plantations in Southeast Asia. This may lead to the generation of PAHs, which can be absorbed by tea leaves [4,26].

Despite its many benefits, studies have also found tea to be contaminated with heavy metals, pesticides and PAHs [27]. Lin et al. (2005) [26] found that during the heating stages of the tea leaves, PAHs are formed. The oxidation process may also help bring out the PAHs developed from the heating processes. Due to the amount of processing black tea undergoes, there has been concern about the formation of PAH in this product. The main aim of this study is to determine the concentration of PAH4 in various black teas available on the Taiwanese market. In order to obtain detection data quickly and effectively while saving costs and facilitating operation, this study adopted the commonly used QuEChERS method to extract PAHs in tea leaves or tea infusions, and analyzed them with HPLC-FLD.

## 2. Materials and Methods

### 2.1. Sample Collection and Preparation

Thirty-four black tea samples were obtained from Taiwanese distributors, tea shops and supermarkets in December 2022. Sixteen of these samples were of Taiwanese origin, the other eleven originated from Vietnam (n = 3), India (n = 6), Indonesia (n = 1), Sri Lanka (n = 5), Myanmar (n = 1), and Kenya (n = 2). The country of origin and sampling location information of black tea samples is presented in Table S1. According to the EU regulations, the performance criteria for PAH4 analysis must be free of interference. The selected representative matrix in this study was freshly picked tea leaves, which were picked in February 2023 at a 1600 m altitude tea plantation (coordinate: 23.607467, 120.709124) in Yunlin county, Taiwan. The preparation of the representative matrix must be carried out in a way that is less likely to cause interferences, in this case, the fresh leaves were steamed.

The conditions of the tea infusion preparation were replicated closely to that of home preparation. Black tea is usually made by brewing 3 g of the tea leaves in 150 mL of hot water for 3 to 5 min. For this study, 2 g (dry weight) of tea leaves were brewed for 3–10 min in 200 mL of 85 °C water. The tea infusions were then left to cool down to room temperature. The cooled tea was then prepared using the QuEChERS technique.

The release of PAH from tea leaves to infusion was calculated using the following equation:

$$Release\left(\%\right)=\frac{C_{t.i}*V_{t.i}}{C_{d.t}*W_{d.t}}$$

C_t.i_: concentration (ng/mL) of PAHs in the tea infusion.
V_t.i_: volume (mL) of the tea infusion.
C_d.t_: concentration (ng/mL) of PAHs in the dry tea.
W_d.t_: weight (g) of dry tea used to prepare the tea infusion.

### 2.2. Extraction and Analysis

#### 2.2.1. Chemicals

Solvents including J.T. Baker^®^ HPLC-grade acetonitrile (ACN) were purchased from Avantor (Radnor, PA, USA); acetic acid and tetrahydrofuran (THF) were purchased from Merck Co. (Darmstadt, Germany). Arium^®^ Pro Ultrapure Lab Water Systems (Sartorius, Goettingen, Germany) was used to prepare distilled deionized water (dd H_2_O). The QuEChERS kits were obtained from Dikma Technologies Co. (Dikma Technologies Inc., Lake Forest, CA, USA). The standards of PAHs (purity) including BaA (99%), BaP (99%), BbF (99%) and CHR (99%) were obtained from AccuStandards Inc. (New Haven, CT, USA)

#### 2.2.2. Extraction of PAHs

The traditional solid phase extraction (SPE) is routinely used in the preparation for the quantification of analytes in biological samples by using cartridges to remove impurities [28]. However, it could reduce the recovery of certain polar compounds and require more time and a higher cost. The QuEChERS method has the same purification effect as the SPE method, but the processing steps are more simplified. Using QuEChERS to extract specific substances in samples is not only convenient but also saves time and extraction solvent usage [29]. Additionally, QuEChERS can improve extraction recovery, data accuracy, and precision [12,30].

In this study, QuEChERS was used to extract PAHs from each sample. For tea leaves, 2 g of the ground sample (dry weight) was placed in a 50 mL centrifuge tube with a ceramic stone and mixed with 10 mL of dd H_2_O; the mixture was then homogenized for 1 min. For tea infusion, 10 mL of the infusion was placed in a 50 mL centrifuge tube with a ceramic stone and homogenized for 1 min. After homogenization, 10 mL of acetonitrile (1% acetic acid) was added and the mixture was homogenized for 1 min. QuEChERS extraction salt packet (4 g magnesium sulfate and 1 g sodium acetate) was added to the mixture and homogenized for 1 min. After the mixture was homogenized and centrifuged at 4000 rpm for 5 min, 6 mL of the supernatant was transferred into a QuEChERS clean up column (450 mg primary secondary amine (PSA), 300 mg C18 EC (Octadecyl siloxane Endcapped), 900 mg magnesium sulfate, and 50 mg of carbon). After the mixture was homogenized and centrifuged at 4000 rpm for 5 min, 1 mL of the supernatant was collected, filtered through a 0.22 µm PVDF membrane, and used for testing.

#### 2.2.3. HPLC Analysis

In our previous study, we used HPLC connected with a fluorescence detector to analyze the content of PAHs in several foodstuffs [31]. HPLC is a popular and easy-to-operate instrument that can effectively reduce experimental costs and obtain accurate results quickly. In order to further improve the Limit of Detection (LOD) and Limit of Quantification (LOQ) of instrument detection, we also referred to the method of Chiang et al. (2021) [16] and still used HPLC but coupled with advanced fluorescence detectors for PAHs analysis.

For the preparation of standard solutions, 10 mg of each standard compound BaP, BaA, BbF, and CHR was accurately weighed and dissolved in ACN and adjusted to 100 mL. Further, 1 mL of each PAH solution above was dissolved in ACN to achieve a total volume of 100 mL, resulting in a concentration of 1 ppm (parts per million) for each compound. The resulting solution was considered the standard stock solution. The stock solutions were stored in a freezer at −20 °C in a light-proof environment. To establish a calibration curve, the standard stock solutions were serially diluted in ACN to obtain concentrations of 0.1, 0.2, 0.5, 0.8, 1, 2, 5, and 10 µg/kg. Mobile phase A comprised distilled deionized water (dd H_2_O). Mobile phase B comprised 960 mL of ACN and 40 mL THF.

The Hitachi HPLC Chromaster system (Hitachi, Tokyo, Japan) for the determination of PAH4 consisted of an HPLC pump (model: 5160), a controller, auto sampler (model: 5260), column oven (model: 5310), and fluorescence detector (model: 5440). The analysis conditions were as follows: Pinnacle II PAH (150 × 3.0 mm, 4µm) (Restek, Bellefonte, PA, USA) kept at 35 °C; mobile phase A (dd H_2_O) and B (ACN:THF, 96:4) (70% B from 0 to 14 min, 90% from 14 to 15 min, 100% from 15 to 27 min, 70% from 27 to 30 min.); flow rate, 1.4 mL/min from 0 to 15 min, 2.0 mL/min from 15 to 27 min, and 1.4 mL/min from 27 to 30 min; injection volume, 20 µL. The excitation (Ex)/emission (Em) wavelengths were as follows: 273 nm/384 nm at 4.396 min (BaA), 273 nm/384 nm at 4.773 min (CHR), 302 nm/452 nm at 6.575 min (BbF), and 290 nm/384 nm at 8.249 min (BaP).

### 2.3. Method Validation

To determine the linearity and range using HPLC, a minimum of five standard solutions with different concentrations (ranging from 0.1 to 10 μg/kg) were required. The selectivity of an analytical method refers to its ability to accurately assess the target analyte without interference from the matrix or other interfering substances. In this study, blank matrix samples of steamed freshly picked tea leaves were chosen, and were analyzed using the specified analytical conditions. It was ensured that the chromatographic peak of the analyte was not affected by the chromatographic peaks of other substances. The accuracy was assessed using 5 determinations over 2 concentration levels for each analyte of interest. Accuracy was determined as the percentage of the mean measured value divided by the true value.

The precision was assessed using 5 determinations over 2 concentration levels for each analyte of interest. Precision is expressed as the percentage of the standard deviation divided by the mean.

$$CV\left(\%\right)=\frac{SD}{mean}*100$$

Mean = the average value, calculated by summing all results and dividing by the total number of results.
SD = the variation in results from the mean.

According to the U.S. Food and Drug Administration [32], the Limit of Detection (LOD) is the lowest quantity of analyte that can be detected but not necessarily quantified. The LOD represents the lowest concentration level that can be distinguished from a blank sample with a specific level of confidence. The LOD of detection is traditionally determined as 3.3 times the signal-to-noise ratio; however, the LOD can also be estimated by dividing the standard deviation of the curve by the slope and multiplying by 3.3. The slope and standard deviation may be obtained using one magnitude of the calibration curve.

$$LOD=3.3(\frac{\sigma }{S})$$

σ: standard deviation.
S: slope.

The Limit of Quantification (LOQ) is the lowest concentration of analyte that can be quantified with acceptable precision and accuracy. The LOQ of detection is traditionally determined as 10 times the signal-to-noise ratio; however, the LOQ can also be estimated by dividing the standard deviation of the curve by the slope and multiplying by 10. The slope and standard deviation may be obtained using one magnitude of the calibration curve.

$$LOQ=10(\frac{\sigma }{S})$$

σ: standard deviation.
S: slope.

## 3. Results

### 3.1. Method Validation

The validation of analytical methods includes linearity and range, specificity, accuracy, precision, LOD and LOQ.

Specificity: As per EU regulations (Commission regulation (EU) No. 836/2011) [33], the performance criteria for the individual PAH4 must be free of interference. Different blank matrices of steamed freshly picked tea leaves were separately spiked with four different concentrations (1, 2, 5, 10 μg/kg) of PAH4. The samples were prepared using the QuEChERS method for extraction and purification, and analysis was performed using HPLC-FLD as described in the Methods Section. The test results showed no interference. The chromatograms of the extracts from steamed *Camellia sinensis* matrices spiked with various concentrations of PAH4 are shown in Figure S1.

Linearity and range: In our study, linearity was determined using regression analysis by plotting experimental preparations of 0.1, 0.2 0.5, 0.8, 1, 2, 5 and 10 µg/kg of standard solutions. A total of eight calibration points were prepared, and chemical analysis was performed using HPLC-FLD. As showed in Table 1, the linear equations (range, 0.1–10 μg/L) for the PAHs were Y = 17,992x + 618.16 (R^2^ = 0.9996) for BaA, Y = 9650x + 415.71 (R^2^ = 0.9996) for CHR, Y = 5327x − 226.41 (R^2^ = 0.9990) for BbF, and Y = 45,574x − 361.09 (R^2^ = 0.9993) for BaP. The correlation coefficient of the linear regression equation for all four PAHs was greater than or equal to 0.999, indicating a good linear relationship. The calibration curve of BaA, CHR, BbF and BaP is shown in Figure S2.

LOD and LOQ: As per EU regulations (Commission regulation (EU) No. 836/2011) [33], the LOD and LOQ for each individual PAH4 must be equal to or lower than 0.30 µg/kg and 0.90 µg/kg, respectively. In this study, the LOD and LOQ for PAH4 were 0.11 and 0.38 for BaA, 0.13 and 0.43 for CHR, 0.13 and 0.42 for BbF, and 0.02 and 0.08 for BaP, respectively. In Table 2, these values for BaA, CHR, BbF, and BAP comply with the LOD and LOQ requirements set forth by the EU regulations, demonstrating that the analytical method used adheres to the specified limits for these PAH compounds.

Accuracy: As shown in Table 1, the recovery rate was assessed by analyzing a blank matrix sample after spiking it with PAH4, and the determined recovery rates ranged from 98% to 112%. These recovery outcomes align with the regulations set by the Taiwan Food and Drug Administration (TFDA) for PAH4 [34], which states recovery rates must be within the range of 50% to 125%. These results indicate that the analysis method used possesses a high level of accuracy.

Precision: Precision was assessed by analyzing a blank matrix sample, and the determined CV% ranged from 0.7% to 4.6%. These outcomes align with the regulations set by the TFDA for PAH4, which states precision values must be below or equal to 32%. These results indicate that the analysis method used possesses a high level of precision.

### 3.2. Effect of Brewing Time on PAH Transfer

**Figure 1:**
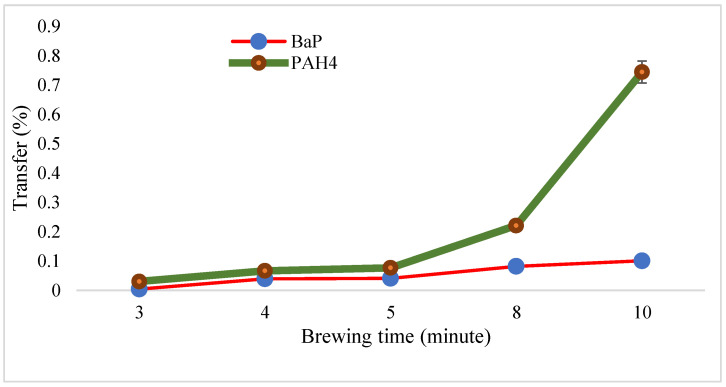
BaP and PAH4 transfer percentage at different brewing periods.

The transfer of PAH4 from tea leaves to tea infusions was found to be time dependent. After 3 min of steeping, the transfer percentage of PAH4 in the tea infusion was determined to be 0.004%. This value increased to 0.04% after 4 min of steeping, indicating a higher transfer rate. Subsequently, at 5 min, the transfer percentage slightly increased to 0.04%, demonstrating a continued transfer of PAH4. After 8 min, the concentration of PAH4 in the tea infusion reached 0.08%, signifying a significant increase in transfer. Finally, at 10 min, the concentration rose to 0.10%, representing the highest observed transfer of BaP (Figure 1).

Similarly, the transfer of PAH4 from tea leaves to tea infusions was also found to be time dependent. After 3 min of steeping, the transfer percentage of PAH4 in the tea infusion was determined to be 0.03%. This value increased to 0.07% after 4 min of steeping, indicating a higher transfer rate. Subsequently, at 5 min, the transfer percentage further increased to 0.08%, demonstrating a continued transfer of PAH4. After 8 min, the concentration of PAH4 in the tea infusion reached 0.22%, signifying a significant increase in transfer. Finally, at 10 min, the concentration rose to 0.74%, representing the highest observed transfer of PAH4 (Figure 1).

### 3.3. Concentration of PAH4 in Tea Leaves

**Figure 2:**
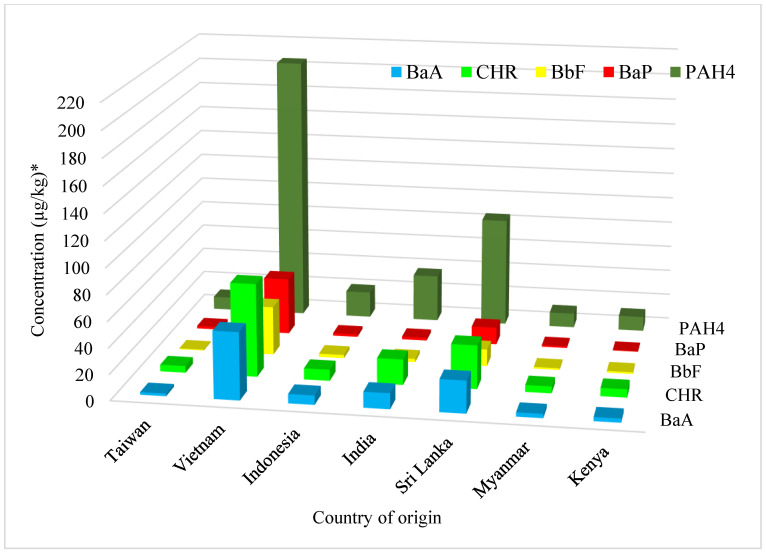
Average concentration of PAH4, BaP, BbF, CHR and BaA in black tea leaves of different countries of origin. * The PAH concentration of all tea leaves is dry weight based.

Table 3 shows that the concentration of PAH4 in black tea leaves ranged from 2.88 µg/kg to 218.21 µg/kg. Among the black teas from all seven countries, those from Vietnam had the highest concentration of BaA, CHR, BbF and BaP in dry teas, with mean concentrations of 51.85 µg/kg, 71.95 µg/kg, 37.73 µg/kg and 44.19 µg/kg, respectively. The value of PAH4 in Vietnamese tea ranged from 193.25 µg/kg to 218.21 µg/kg, with a mean content of 205.73 µg/kg. The teas with the second highest concentration were the teas which originated from Sri Lanka. The content of PAH4 in Sri Lankan teas ranged from 63.63 µg/kg to 97.81 µg/kg, with a mean of 85.00 µg/kg. The average concentrations of BaA, CHR, BbF and BaP were 24.88 µg/kg, 33.63 µg/kg, 13.05, and 13.44 µg/kg, respectively. The lowest concentration of PAH4 was determined in teas which originated in Taiwan. The sum of all four PAHs ranged from 2.88 µg/kg to 40.76 µg/kg, with a mean sum of 10.12 µg/kg. The means of BaA, CHR, BbF and BaP were 2.06 µg/kg, 5.20 µg/kg, 0.66 µg/kg, and 2.10 µg/kg, respectively. BbF was not detected in many of the Taiwanese tea samples (Table 3 and Figure 2).

Through further comparison between Table 3 and Figure 2, it can be found that the average concentrations of PAH4, BaA, CHR, BbF and BaP in black tea leaves from different countries are all highest in tea from Vietnam, followed by tea from Sri Lanka, with tea from India ranking third, and tea from Taiwan fourth. The tea from Indonesia, Kenya and Myanmar was not compared because the number of samples was insufficient.

### 3.4. Concentration of PAH4 in Tea Infusions

This study analyzed the tea infusions of the previously studied tea leaves, as well as bottled infusions and infusions from tea shops. Among the teas analyzed, the infusions from India had the highest concentration of PAH4, with a mean concentration of 0.08 µg/kg. This was followed by the teas from Vietnam, with a mean concentration of 0.07 µg/kg. Sri Lanka followed with the third highest concentration of PAH4, with an average concentration of 0.05 µg/kg. The mean concentration of PAH4 in the teas which originated from Taiwan was 0.02 µg/kg. The concentration of PAH4 in all teas originating from Indonesia, Kenya, and Myanmar was determined to be below the LOD (Table S2).

Similar to the tea leaves, the tea infusions had higher concentrations of BaA and CHR compared to BbF and BaP. The concentrations of BaA and CHR ranged from ND to 0.21 µg/kg and ND to 0.24 µg/kg, respectively. The concentrations of BaP ranged from ND to 0.05 µg/kg. The concentration of BbF was determined to be below the LOD in all infusions analyzed. The concentrations of BaA, CHR and BaP were determined to be below the LOD in the majority of the infusions analyzed (Table S2).

This study also determined the transfer percentage of PAH4 from tea leaves to tea infusions, whereby transfer percentages are shown in Table S2. BaA was determined to have the highest transfer, with transfer percentages ranging from ND to 47.13%. This was followed by CHR, with transfer percentages ranging from ND to 29.82%. BaP followed with ranges from ND to 0.37%. BbF was not detected in any of the samples analyzed.

## 4. Discussion

### 4.1. Effect of Brewing Time on PAH Transfer

The results of this study indicate that the transfer of PAH4 from tea leaves to tea infusions is influenced by the duration of steeping. The gradual increase in PAH4 concentration observed with longer steeping times suggests that PAH4 compounds present in tea leaves leach into hot water during the infusion process. This phenomenon is likely due to the heat and liquid facilitating the extraction of PAH4 from the tea leaves, leading to higher concentrations in the resulting infusions.

The initial low concentration of PAH4 at 3 min suggests that minimal transfer occurs within the first few minutes of steeping. However, as the steeping time progresses, the concentration of PAH4 in the tea infusions steadily rises. This could be attributed to the prolonged contact between the tea leaves and hot water, enabling a more thorough extraction of PAH4 compounds.

Based on the results, if an individual wants to actively reduce their PAH intake from tea, one approach may be to minimize the brewing time. The findings indicate that PAH concentrations in tea infusions are influenced by the brewing process, with shorter brewing times resulting in lower levels of PAHs. Therefore, by brewing tea leaves for the shortest amount of time necessary to achieve the desired flavor, individuals can potentially reduce their PAH intake from tea.

### 4.2. PAH Concentration in Tea Leaves

The total mean concentration of PAH4 in tea leaves ranged from 2.88 µg/kg to 218.2 µg/kg. The highest concentration was found in teas originating from Vietnam, with an average PAH4 concentration of 205.7 µg/kg. Sri Lanka followed with the second highest concentration, with a mean PAH4 concentration of 85 µg/kg. The teas with the lowest concentration of PAH4 were the teas from Taiwan, Myanmar and Kenya, with average concentrations of 10.12 µg/kg, 11.23 µg/kg and 11.40 µg/kg, respectively.

Despite the fact that all the teas examined in this study were categorized as black teas, a substantial variation was observed in the concentrations of PAHs among teas originating from different countries. These differences can be attributed to both the tea-processing techniques employed and environmental contamination. While the primary factor contributing to the variations is likely the processing methods used, the specific details of the processing techniques applied to the teas under investigation remain unknown. Environmental pollution could potentially influence PAH levels to some extent, although the magnitude of its effect is expected to be smaller compared to that of processing. Notably, a study conducted in Vietnam revealed elevated levels of atmospheric PAHs. This study determined the atmospheric levels of PAH to be 63 ng/m^3^ on average [35]. In comparison, a study conducted in Taichung, Taiwan, the atmospheric levels of PAH were determined to range from 7.29 to 26 ng/m^3^, providing further evidence of the potential influence of environmental factors [36].

**Figure 3:**
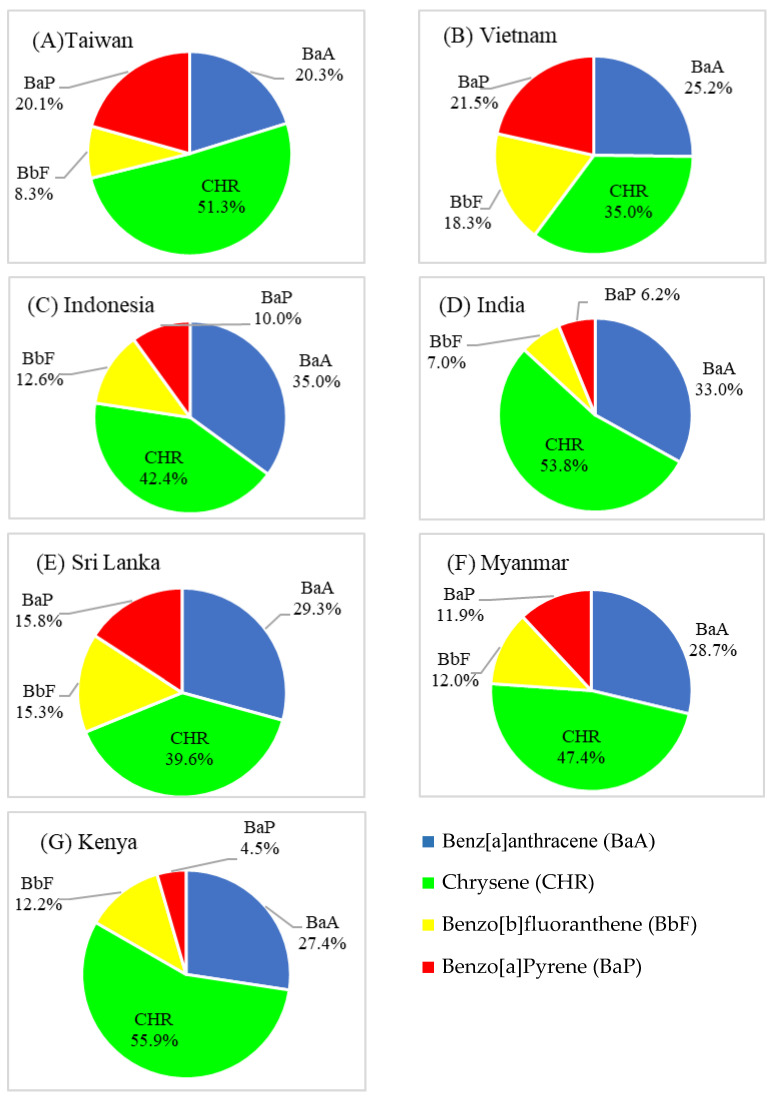
Average distribution of BaA, CHR, BbF and BaP in black tea leaves of different countries of origin. (**A**) Taiwan, (**B**) Vietnam, (**C**) Indonesia, (**D**) India, (**E**) Sri Lanka, (**F**) Myanmar, (**G**) Kenya.

The average distribution of BaA, CHR, BbF and BaP in black tea leaves from different origins is shown in Figure 3. BaP in black tea leaves from Kenya and India has lower levels among the four PAHs. CHR exhibited the highest concentration among the four PAHs analyzed, accounting for 35.0% to 55.9% of the total sum of PAH4. Following closely, BaA represented the second highest concentration, comprising 20.3% to 35.0% of PAH4. In contrast, BbF and BaP exhibited lower concentrations compared to CHR and BaA. These findings align with the results reported by Phan Thi et al. (2020) [37], where higher concentrations of CHR and BaA were observed relative to those of BbF and BaP. Notably, BaA and CHR belong to the four-ringed PAHs with molecular weights of 228 g/mole, while BbF and BaP are five-ringed PAHs with molecular weights of 252 g/mole. According to Huang and Penning (2014) [27], smaller molecules of PAHs (BaA and CHR) are formed at a higher rate compared to larger molecules of PAHs (BbF and BaP), as the latter require the incorporation of additional benzene rings, which introduces a kinetic constraint on their formation. Phan Thi et al. (2020) [37] also indicated that the mean PAH4 concentration of black teas (54.3 µg/kg, n = 4) was remarkably higher than that of herbal teas (16.4 µg/kg, n = 5), green teas (14.2 µg/kg, n = 7) and oolong teas (6.6 µg/kg, n = 8). Moreover, the highest (35.8 µg/kg) and average (10.8 µg/kg) concentrations of BaP also appeared in black tea samples. In addition, the concentration ratio of each PAH (BaA = 23.6%, CHR = 33.2%, BbF = 23.3%, BaP = 19.9%) is close to that of black tea leaves from Vietnam in this study (BaA = 25.2%, CHR = 35.0%, BbF = 18.3%, BaP = 21.5%) (Figure 3).

To date, some of the tea production areas in Asia still use traditional or obsolete equipment for drying tea leaves. From the results of this study, it can be preliminarily identified that the higher PAH4 and BaP values in black tea are related to the drying equipment, heat source and temperature control of tea manufacturing. Especially, the extensive use of wood or charcoal firing as a heat source is most likely to generate the PAHs. These PAHs can be absorbed by surface of tea leaves [4,5]. Further studies could collect more commercial black tea leaves with various countries of origin and focus in depth on the factors that influence the concentration of PAHs in tea leaves, and explore the relationship between the formation of specified PAHs and manufacturing conditions such as temperature, humidity, time, heat source or combustion materials.

## 5. Conclusions

In this study, the laboratory operations showed that a tea sample only takes approximately 2 h from pretreatment and QuEChERS extraction to HPLC-FLD analysis and data acquisition. The LOD and LOQ for BaA, CHR, BbF and BaP were 0.02–0.11 µg/kg and 0.08–0.38 µg/kg, respectively, which are within the regulations set by the EU. The method validation results demonstrated that our approach was accurate, and precise, ensuring the reliability of the subsequent findings. This study identified varying differences among black teas of different origins. Notably, black tea originating from Vietnam had the highest concentration of PAH4 among all countries, warranting attention to the production and processing methods of black tea in that region. In comparison, teas originating from Taiwan had less PAH4. Black tea infusions were shown to have significantly fewer PAHs compared to tea leaves. The brewing of the tea leaves in water allowed for only small amounts of PAH4 to transfer into the infusion. Currently, there are no guidelines for BaP or PAH4 in tea products. This study opens up an opportunity for regulatory bodies to begin to implement standards and guidelines for PAHs in tea products.

## Figures and Tables

**Table 1:** The linearity, correlation coefficient (r^2^) of PAH4, recoveries (%) and CV% for extraction of the spiked PAH4 in steamed *Camellia sinensis* leaves’ matrices.

PAH	Retention Time (min) *	Linear Regression Equation **	r^2^	Mean Recovery (%) (CV%) (n = 5)	
				1.0 µg/kg	5.0 µg/kg
BaA	4.396	Y = 17,992X + 618.16	0.9996	98 (2.6)	105 (2.4)
CHR	4.773	Y = 9650X + 415.71	0.9996	100 (0.7)	108 (4.6)
BbF	6.575	Y = 5327X − 226.41	0.9990	100 (4.0)	109 (3.1)
BaP	8.249	Y = 45,574X − 361.09	0.9993	100 (1.8)	112 (2.7)

* Reference Figure S1 for each PAH compound. ** Linear range: 0.1–10 µg/kg.

**Table 2:** Limit of detection (LOD) and limit of quantification (LOQ) for BaA, CHR, BbF and BaP in this study.

	BaA	CHR	BbF	BaP	EU Regulation
	(µg/kg)				
LOD	0.11	0.13	0.13	0.02	≤0.3
LOQ	0.38	0.43	0.42	0.08	≤0.9

**Table 3:** Content of PAH4 in black tea leaves sourced from various countries.

Country	Sample Number	Concentration (µg/kg) ^2^				
		BaA	CHR	BbF	BaP	PAH4
Taiwan	1	2.63	7.18	0.84	4.40	15.05
	2	0.68	1.17	ND	5.11	7.03
	3	0.98	2.91	ND	0.86	4.81
	4	1.04	5.07	ND	0.48	6.66
	5	ND ^1^	6.54	ND	2.95	10.11
	6	1.23	1.29	ND	0.29	2.88
	7	0.89	2.57	ND	1.61	5.13
	8	0.93	3.16	ND	ND	4.17
	9	ND	4.02	ND	ND	4.64
	10	11.66	18.04	ND	5.34	40.76
	Mean	2.06	5.20	0.66	2.10	10.12
Vietnam	17	56.74	78.24	35.31	47.92	218.2
	18	46.97	65.67	40.15	40.47	193.3
	Mean	51.85	71.95	37.73	44.19	205.8
India	20	6.79	8.00	1.57	0.85	17.21
	21	10.46	10.85	4.22	3.69	29.22
	24	19.07	40.20	1.87	2.25	63.39
	Mean	12.10	19.68	2.55	2.27	36.61
Indonesia	26	7.14	8.62	2.57	2.03	20.36
Kenya	28	3.12	6.37	1.39	0.51	11.40
Sri Lanka	29	17.85	25.36	9.40	11.02	63.63
	30	28.50	39.08	14.32	11.66	93.56
	33	28.29	36.46	15.41	17.64	97.81
	Mean	24.88	33.63	13.05	13.44	85.00
Myanmar	34	3.23	5.32	1.34	1.34	11.23

^1^ ND = Not detected, <LOD. ^2^ The PAH concentration of all tea leaves is dry weight based. Reference Table S1 for sample number and origin.

## Data Availability

Data are contained within the article and Supplementary Materials.

## Supplementary Materials

The following supporting information can be downloaded at: https://www.mdpi.com/article/10.3390/toxics12020148/s1. Figure S1: Chromatograms of the extracts from steamed *Camellia sinensis* matrices spiked with various concentrations of PAH4; Figure S2: HPLC analysis calibration curve of BaA, CHR, BbF and BaP in this study; Table S1: country of origin and sampling location of black tea samples; Table S2: concentration of PAH4 in black tea infusions from various countries.
