# Supplementary material for: Using QuEChERS and HPLC Method to Monitor the Background Concentration of Polycyclic Aromatic Hydrocarbons in Commercial Black Tea Leaves and Infusions in Taiwan
Source: Toxics. 2024 Feb 14;12(2):148. doi: 10.3390/toxics12020148 (PMC10893135; doi:10.3390/toxics12020148)
Supplement: Supplementary file 1 [file toxics-12-00148-s001.zip › toxics-2843018-supplementary.pdf]

Supplementary Materials: The following supporting information can be downloaded at: www.mdpi.com/xxx/s1

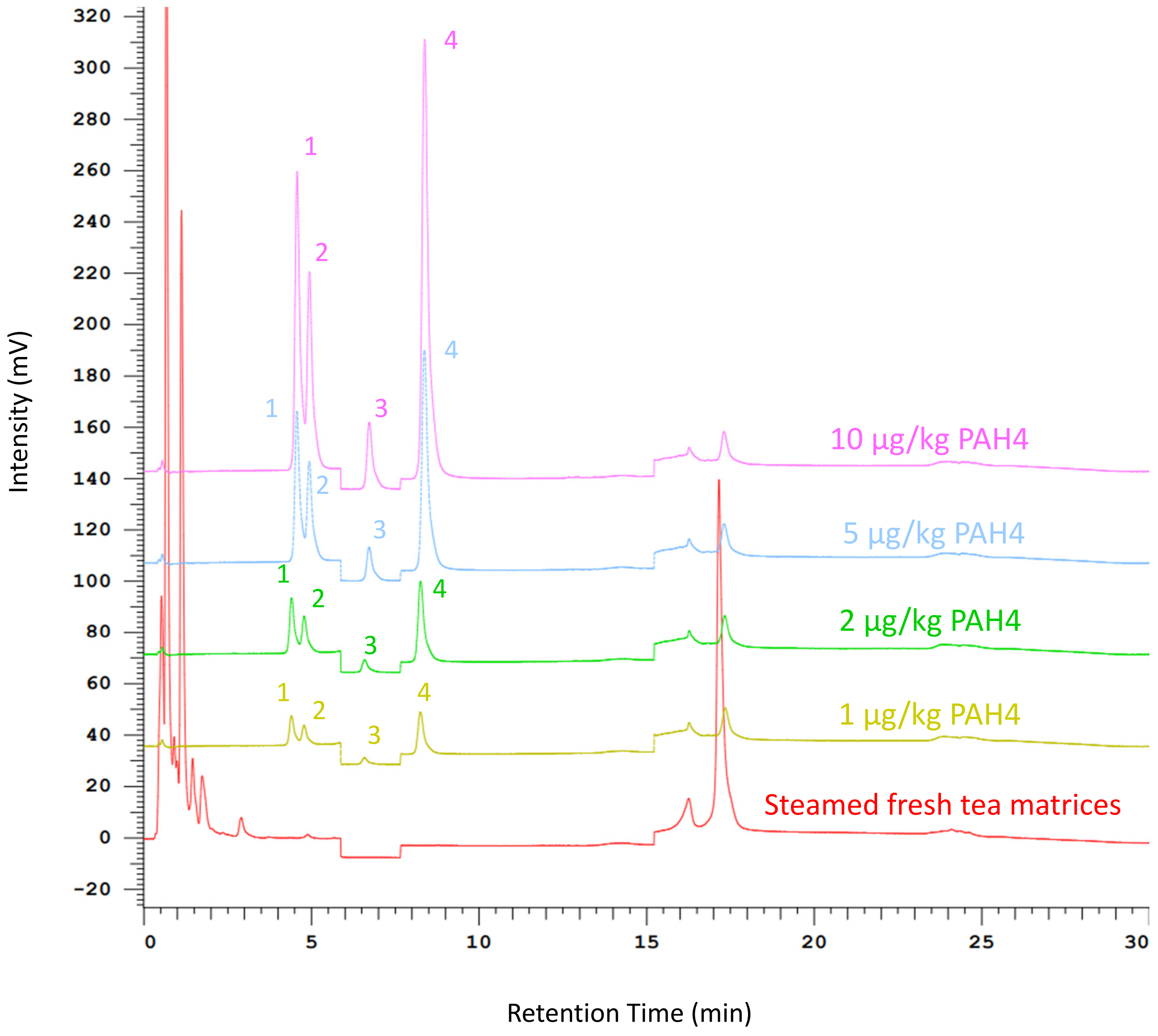

Figure S1. Chromatograms of the extracts from steamed *Camellia sinensis* matrices spiked with various concentrations of PAH4. Peak: 1: Benzo[a]anthracene (BaA), 2: Chrysene (CHR), 3: Benzo[b]fluoranthene (BbF), 4: Benzo[a]Pyrene (BaP).

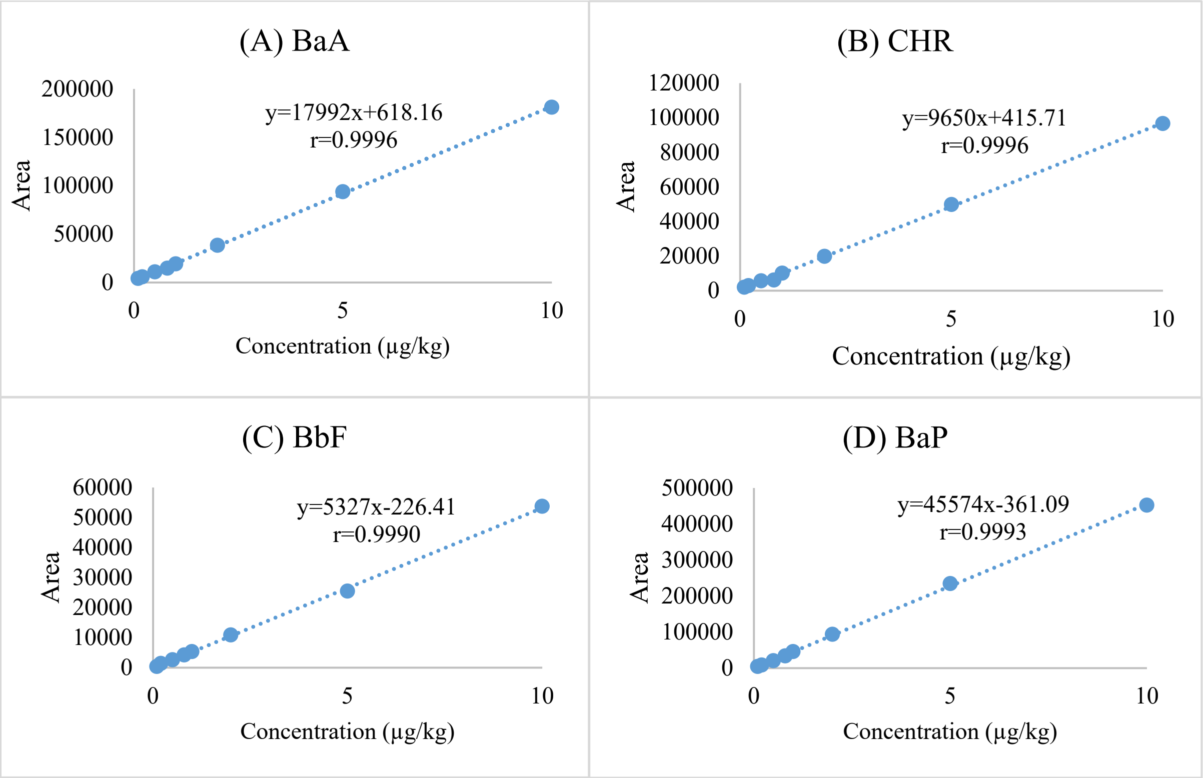

Figure S2. HPLC analysis calibration curve of BaA, CHR, BbF and BaP in this study.

Table S1. Country of origin and sampling location of black tea samples.

Sample Number	Country of Origin	Group	Location
1	Taiwan	Loose Leaf	Factory
2	Taiwan	Loose Leaf	Factory
3	Taiwan	Loose Leaf	Factory
4	Taiwan	Loose Leaf	Factory
5	Taiwan	Loose Leaf	Factory
6	Taiwan	Loose Leaf	Supermarket
7	Taiwan	Loose Leaf	Supermarket
8	Taiwan	Loose Leaf	Factory
9	Taiwan	Loose Leaf	Supermarket
10	Taiwan	Teabag	Supermarket
11	Taiwan	Bottle	Supermarket
12	Taiwan	Bottle	Supermarket
13	Taiwan	Bottle	Supermarket
14	Taiwan	Bottle	Supermarket
15	Taiwan	Tea shop	Tea shop
16	Taiwan	Tea shop	Tea shop
17	Vietnam	Loose Leaf	Factory
18	Vietnam	Loose Leaf	Factory
19	Vietnam	Tea shop	Tea shop
20	India	Loose Leaf	Factory
21	India	Loose Leaf	Factory
22	India	Bottle	Supermarket
23	India	Bottle	Supermarket
24	India	Loose Leaf	Supermarket
25	India	Bottle	Supermarket
26	Indonesia	Loose Leaf	Factory
27	Kenya	Tea shop	Tea shop
28	Kenya	Loose Leaf	Factory
29	Sri Lanka	Loose Leaf	Factory
30	Sri Lanka	Loose Leaf	Factory
31	Sri Lanka	Tea shop	Tea shop
32	Sri Lanka	Bottle	Supermarket
33	Sri Lanka	Loose Leaf	Supermarket
34	Myanmar	Teabag	Supermarket

Table S2. Concentration of PAH4 in black tea infusions from various countries.

Country	Sample number	Concentration(μg/kg)					Transfer (%)				
		BaA	CHR	BbF	BaP	PAH4	BaA	CHR	BbF	BaP	PAH4
Taiwan	1	ND[1]	ND	ND	ND	ND	0.00	0.00	0.00	0.00	0.00
	2	ND	ND	ND	ND	ND	0.00	0.00	0.00	0.00	0.00
	3	ND	ND	ND	ND	ND	0.00	0.00	0.00	0.00	0.00
	4	ND	ND	ND	ND	ND	0.00	0.00	0.00	0.00	0.00
	5	ND	ND	ND	ND	ND	0.00	0.00	0.00	0.00	0.00
	6	0.12	ND	ND	ND	0.12	47.13	0.00	0.00	0.00	20.12
	7	ND	ND	ND	ND	ND	0.00	0.00	0.00	0.00	0.00
	8	ND	ND	ND	ND	ND	0.00	0.00	0.00	0.00	0.00
	9	ND	0.24	ND	ND	0.29	0.00	29.82	0.00	0.00	25.81
	10	ND	ND	ND	ND	ND	0.00	0.00	0.00	0.00	0.00
	11	ND	ND	ND	ND	ND	-	-	-	-	-
	12	ND	ND	ND	ND	ND	-	-	-	-	-
	13	ND	ND	ND	ND	ND	-	-	-	-	-
	14	ND	ND	ND	ND	ND	-	-	-	-	-
	15	ND	ND	ND	ND	ND	-	-	-	-	-
	16	ND	ND	ND	ND	ND	-	-	-	-	-
	Mean	0.01	0.01	ND	ND	0.02	4.71	2.98	0.00	0.00	4.59
Vietnam	17	0.13	ND	ND	ND	0.13	1.44	0.00	0.00	0.00	0.35
	18	ND	ND	ND	0.04	0.04	0.00	0.00	0.00	0.37	0.08
	19	ND	ND	ND	0.03	0.03	-	-	-	-	-
	Mean	0.04	ND	ND	0.02	0.07	0.72	0.00	0.00	0.18	0.22

[1]ND= Not Detected, <LOD.

Reference table S1 for sample number and origin.

Table S2. (continued) Concentration of PAH4 in black tea infusions from various countries.

		Concentration (µg/kg)					Transfer (%)				
		**BaA**	**CHR**	**BbF**	**BaP**	**PAH4**	**BaA**	**CHR**	**BbF**	**BaP**	**PAH4**
India	20	ND	ND	ND	ND	ND	0.00	0.00	0.00	0.00	0.00
	21	ND	ND	ND	ND	ND	0.00	0.00	0.00	0.00	0.00
	22	ND	ND	ND	ND	ND	-	-	-	-	-
	23	ND	ND	ND	ND	ND	-	-	-	-	-
	24	0.21	0.24	ND	ND	0.45	5.55	3.01	0.00	0.00	3.58
	25	ND	ND	ND	ND	ND	-	-	-	-	-
	Mean	0.04	0.04	ND	ND	0.08	1.85	1.00	0.00	0.00	1.19
Indonesia	26	ND	ND	ND	ND	ND	0.00	0.00	0.00	0.00	0.00
Kenya	27	ND	ND	ND	ND	ND	-	-	-	-	-
	28	ND	ND	ND	ND	ND	0.00	0.00	0.00	0.00	0.00
	Mean	ND	ND	ND	ND	ND	0.00	0.00	0.00	0.00	0.00
Sri Lanka	29	ND	ND	ND	ND	ND	0.00	0.00	0.00	0.00	0.00
	30	ND	ND	ND	0.05	0.05	0.00	0.00	0.00	2.29	0.29
	31	ND	ND	ND	0.03	0.03	-	-	-	-	-
	32	0.12	ND	ND	0.03	0.15	-	-	-	-	-
	33	ND	ND	ND	ND	ND	0.00	0.00	0.00	0.00	0.00
	Mean	0.02	ND	ND	0.02	0.05	0.00	0.00	0.00	0.76	0.10
Myanmar	34	ND	ND	ND	ND	ND	0.00	0.00	0.00	0.00	0.00
